# Voice Prosthetic Biofilm Formation and *Candida* Morphogenic Conversions in Absence and Presence of Different Bacterial Strains and Species on Silicone-Rubber

**DOI:** 10.1371/journal.pone.0104508

**Published:** 2014-08-11

**Authors:** Henny C. van der Mei, Kevin J. D. A. Buijssen, Bernard F. A. M. van der Laan, Ekatarina Ovchinnikova, Gésinda I. Geertsema-Doornbusch, Jelly Atema-Smit, Betsy van de Belt-Gritter, Henk J. Busscher

**Affiliations:** 1 University of Groningen and University Medical Center Groningen, Department of Biomedical Engineering, Groningen, the Netherlands; 2 University of Groningen and University Medical Center Groningen, Department of Otorhinolaryngology and Head and Neck Surgery, Groningen, the Netherlands; Ghent University, Belgium

## Abstract

Morphogenic conversion of *Candida* from a yeast to hyphal morphology plays a pivotal role in the pathogenicity of *Candida* species. Both *Candida albicans* and *Candida tropicalis*, in combination with a variety of different bacterial strains and species, appear in biofilms on silicone-rubber voice prostheses used in laryngectomized patients. Here we study biofilm formation on silicone-rubber by *C. albicans* or *C. tropicalis* in combination with different commensal bacterial strains and *lactobacillus* strains. In addition, hyphal formation in *C. albicans* and *C. tropicalis*, as stimulated by *Rothia dentocariosa* and *lactobacilli* was evaluated, as clinical studies outlined that these bacterial strains have opposite results on the clinical life-time of silicone-rubber voice prostheses. Biofilms were grown during eight days in a silicone-rubber tube, while passing the biofilms through episodes of nutritional feast and famine. Biofilms consisting of combinations of *C. albicans* and a bacterial strain comprised significantly less viable organisms than combinations comprising *C. tropicalis*. High percentages of *Candida* were found in biofilms grown in combination with *lactobacilli*. Interestingly, *L. casei*, with demonstrated favorable effects on the clinical life-time of voice prostheses, reduced the percentage hyphal formation in *Candida* biofilms as compared with *Candida* biofilms grown in absence of bacteria or grown in combination with *R. dentocariosa*, a bacterial strain whose presence is associated with short clinical life-times of voice prostheses.

## Introduction

**Figure 1:**
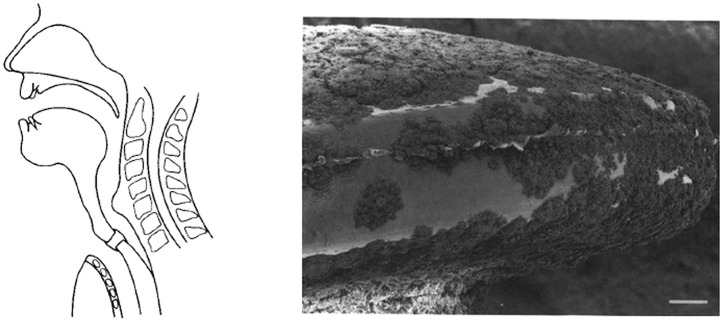
Restoration of voice after total laryngectomy using a tracheostoma valve. Schematic drawing of voice restoration after laryngectomy using a tracheostoma (left) and SEM of a mixed species (yeast and bacteria) biofilm formation on the tracheostoma valve after insertion for 40 days into a patient (right, bar marker indicates 600 µm). Taken with permission from [7].

Verbal communication in patients after laryngectomy can be restored (Figure 1) by placing a silicone-rubber voice prosthesis into a surgically created puncture between the trachea and esophagus [1]. By closing the stoma with a finger, air from the lungs can be forced through the silicone-rubber valve to produce tissue vibration and accompanying voice. In the non-sterile environment of the esophagus, valves sides of voice prostheses become rapidly colonized by microorganisms, leading to increased airflow resistance or leakage of food and liquid [2], which results in frequent replacements of prostheses. *Candida albicans* and *Candida tropicalis* are regarded as the main fungal species in voice prosthetic biofilms, and seldom exist alone in a biofilm. Next to *Candida* species, several other bacterial members of the commensal oral and skin flora of the host have been detected in voice prosthetic biofilms [1], [3], mainly comprising streptococci, staphylococci and *lactobacilli*. Clearly, these commensal have easy access to prosthesis site. Recently it was shown that the diversity (i.e. number of bacterial species detected) of pathogens detected on voice prostheses correlated positively with the diversity of pathogens in the oral cavity [4].

An important aspect of *Candida* pathogenicity [5] is the morphogenic conversion between yeast and filamentous forms of hyphal growth [6]. Hyphae have been associated with the invasive characteristics of *Candida* species and their growth into silicone-rubber, when exposed to cycles of feast and famine [7]. A mature biofilm on a voice prosthetic surface consists of densely packed *Candida* and bacteria, embedded within a matrix of exopolysaccharides [8] with a high degree of interspecies interaction [9] and co-adhesion occurring on a spatio-temporal basis. The presence of bacteria is essential for the integrity of voice prosthetic biofilms [10] and like in the oral cavity, metabolic cooperation among the organisms may be of importance to the establishment of stable biofilm communities [11]. Interestingly, combinations of different bacterial strains, like *Rothia dentocariosa* with *C. albicans* have been associated with a clinical life-time of voice prostheses lower than average, while combinations with *lactobacilli* have been suggested to prevail in prostheses with extended clinical life-times [1], [3]. It is currently unknown why certain combinations of bacteria with *Candida* are more or less harmful than others.

The aim of this study was to investigate early biofilm formation on silicone-rubber by combinations of *Candida* (*C. albicans* or *C. tropicalis*) with different commensal bacterial strains and strains of *lactobacilli*. In addition, hyphal formation in *C. albicans* and *C. tropicalis*, as stimulated by *R. dentocariosa* or *lactobacilli* was evaluated, as clinical studies outlined that these bacterial strains have opposite results on the life-time of silicone-rubber voice prostheses in vivo [1], [3]. All strains and species used in this study, except the *lactobacilli*, were originally isolated from biofilms on explanted voice prostheses.

## Materials and Methods

### Silicone-rubber

Commercially available silicone-rubber tubes (inner diameter 4.0 mm) were used (Rubber B.V., Hilversum, The Netherlands). Silicone-rubber surfaces were characterized by water contact angles, taken at 25°C using the sessile drop (3 µL) technique and a homemade counter monitor. This monitor registers the contour of a liquid droplet based on grey value thresholding, after which contact angles are calculated from the height and base width of a droplet.

### Biofilm Formation

Two yeast strains (C. albicans GBJ 13/4A and C. tropicalis GB 9/9) and seven bacterial strains (Staphylococcus aureus GB 2/1, Staphylococcus epidermidis GB 9/6, Streptococcus salivarius GB 24/9, R. dentocariosa GBJ 52/2B, Lactobacillus casei ATCC 393, Lactobacillus acidophilus ATCC 4356 and Lactobacillus crispatus ATCC 33820) were selected for this study. All strains, except for the lactobacilli, were clinical isolates from biofilms on voice prosthesis. Yeast and bacteria were cultured in a mixture of 30% brain heart infusion broth (OXOID, Basingstoke, UK) and 70% defined yeast medium (per liter: 7.5 g glucose, 3.5 g (NH_4_)_2_SO_4_, 1.5 g L-asparagine, 10 mg L-histidine, 20 mg DL-methionine, 20 mg DL-tryptophane, 1 g KH_2_PO_4_, 500 mg MgSO_4_.7H_2_O, 500 mg NaCl, 500 mg CaCl_2_.2H_2_O, 100 mg yeast extract, 500 µg H_3_BO_3_, 400 µg ZnSO_4_.7H_2_O, 120 µg Fe(III)Cl_3_, 200 µg Na_2_MoO_4_.2H_2_O, 100 µg KI, 40 µg CuSO_4_.5H_2_O). Each strain was grown overnight at 37°C for 24 h. Subsequently, cultures of a yeast and bacterial strain were mixed in one to one volume ratio and comprised approximately 3×10^7^ yeast per mL and 2×10^9^ bacteria per mL. The resulting dual species suspension was used for biofilm growth (see also Table 1).

**Table 1:** Total number of CFU/cm^2^ (*Candida* and bacteria) grown on silicone-rubber in an eight day time period after inoculation with a combination of *C. albicans* or *C. tropicalis* and a commensal bacterial strain or a *lactobacillus* strain, together with the percentage prevalence of the yeast in the final biofilm.

*C. albicans* combined with	Total CFUs (10^5^/cm^2^)	*C. albicans* (%)
No bacteria	45.5±20.6^a^	100^a^
*Rothia dentocariosa*	4.4±0.4^b^	1.1±0.2^b^
*Streptococcus salivarius*	3.5±0.3^c^	15.7±2.0^c^
*Staphylococcus epidermidis*	17.0±1.3^d^	3.2±0.1^d^
*Staphylococcus aureus*	0.2±0.1^e^	3.4±2.6^b,d^
*Lactobacillus casei*	5.0±0.5^b^	45.4±6.8^e^
*Lactobacillus acidophilus*	1.3±0.3^f^	74.7±25.9^f^
*Lactobacillus crispatus*	0.9±0.1^f^	36.5±6.1^e^

*C. tropicalis* combined with	CFU (10^5^/cm^2^)	*C. tropicalis* (%)
No bacteria	19±8^a^	100^a^
*Rothia dentocariosa*	50±5^b^	0.3±0.1^b^
*Streptococcus salivarius*	9±1^a^	0.6±0.1^c^
*Staphylococcus epidermidis*	47±3^b^	5.4±0.5^d^
*Staphylococcus aureus*	280±70^c^	0.3±0.1^b^
*Lactobacillus casei*	200±30^c^	27.2±6.1^e^
*Lactobacillus acidophilus*	62±10^b,d^	8.9±2.1^f^
*Lactobacillus crispatus*	15±3^a^	34.9±10.5^e^

During the growth period, biofilms were exposed to nutritional feast and famine. All results are from triplicate experiments with separate microbial cultures and are presented ± SD. a ≠ b ≠ c ≠ d ≠ e ≠ f at p<0.05 analyzed per column and separately for *C. albicans* and *C. tropicalis*.

Biofilms were grown in silicone-rubber tubes and maintained at a temperature between 36°C and 37°C during an experiment [1]. To grow biofilms, the silicone-rubber tubes were filled for 5 h with the dual species suspension.

**Figure 2:**
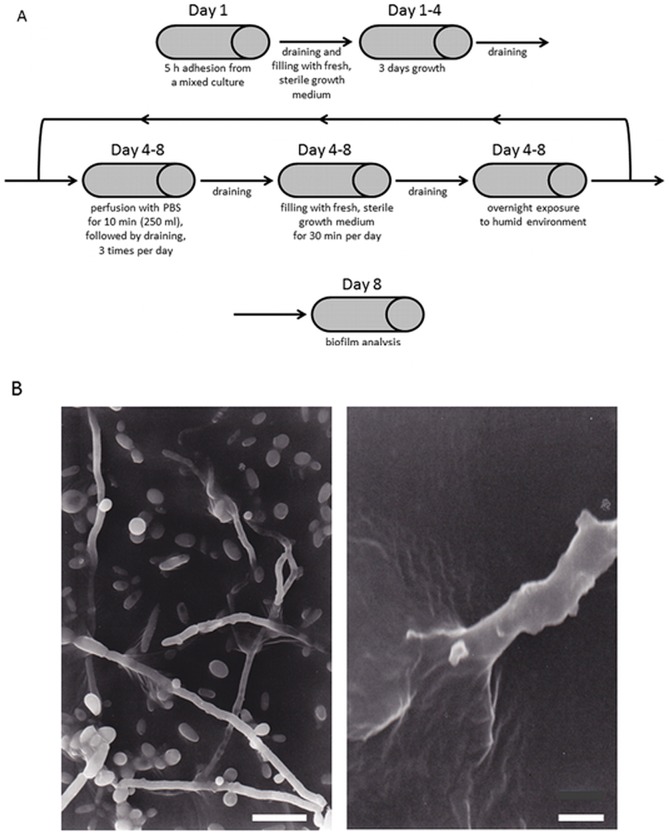
Flow diagram of the feast and famine cycle and resulting hyphal ingrowth. A. Time sequence of biofilm formation in silicone rubber tubes from mixed cultures of *Candida* species and a bacterial strain during exposure to feast and famine. B. Scanning electron micrograph of *C. tropicalis* hyphae penetrating into silicone-rubber after 12 days growth under conditions of feast and famine. Bar markers represent 10 µm and 2.5 µm for the left and right panel, respectively. Taken with permission from [7].

After 5 h, adhering bacteria and yeast were allowed to grow into a biofilm on the silicone-rubber tubes during three days, by filling the tubes with growth medium. From day four till day seven, the tubes were perfused three times a day with 250 mL phosphate buffered saline (10 mM potassium phosphate and 150 mM sodium chloride, pH 6.8). Subsequently, the tubes were left in the moist environment. At the end of each day, the tubes were filled with growth medium during 30 min and left overnight in the moist environment of the drained tubes (see Figure 2A). This cycle of feast and famine was performed in order to mimic the pattern of eating and drinking of a patient and found necessary to stimulate ingrowth of hyphae into the silicone rubber (see also Figure 2B).

### Evaluation of Biofilms: Amount of Biofilm

On day eight of the experiment, the silicone-rubber tubes were removed to assess the number of colony forming yeast and bacteria [1]. To this end, tubes were cut open and biofilms were removed by cotton swabs and sonication in Reduced Transport Fluid (per liter: NaCl 0.9 g, (NH_4_)_2_SO_4_ 0.9 g, KH_2_PO_4_ 0.45 g, Mg_2_SO_4_ 0.19 g, K_2_HPO_4_ 0.45 g, EDTA 0.37 g, L-Cysteine HCl 0.2 g, pH 6.8), after which the resulting suspension was serially diluted and plated on MRS (de Man, Rogosa and Sharpe) agar for yeast and blood agar for bacteria. Plates were incubated at 37°C in an aerobic incubator for 3 days prior to enumeration. In case of combinations with *lactobacilli*, the resulting suspension was plated on MRS plates with incubation in 5% carbon dioxide incubator, while *Candida* strains were plated on Sabouraud agar plates (incubation at 26°C).

In order to demonstrate the presence of a biofilm in the silicone-rubber tubes, biofilms were imaged using optical coherence tomography (OCT) and confocal laser scanning microscopy (CLSM). OCT allowed in situ imaging of biofilms within the silicone-rubber tubes without additional staining or sectioning using a spectral domain OCT (Ganymade, Thorlabs Inc., Munich, Germany) with an axial resolution of 5.8 µm and a lateral resolution of 8 µm. CLSM images were collected using a Leica TCS-SP2 CSLM (Leica Microsystems Heidelberg GmbH, Heidelberg, Germany) after taking transversal sections from the silicone-rubber tube. Sections were placed on a microscope glass slide and biofilm stained by live/dead stain (*Bac*light™, Molecular probes Europe BV) for 30 min in the dark, after which images were taken.

### Evaluation of Biofilms: Hyphal Induction by Selected Bacterial Strains

Biofilms grown with combinations of *Candida* and *R. dentocariosa* or *lactobacilli*, were further examined immediately after suspending of the biofilm in RTF. The resulting microbial suspension was analyzed using phase-contrast microscopy in order to determine the percentage of fungi in a yeast or hyphal morphology after observing 500 *Candida* cells in total in one experiment.

### Statistical Analysis

All experiments were done in triplicate and data were compared using a Student t-test, accepting p<0.05 as statistically significant.

## Results

Water contact angles on the silicone-rubber tubing amounted 110±1 degrees, attesting to the hydrophobic nature of the material and corresponding with water contact angles on medical-grade silicone-rubber voice prostheses [12].

**Figure 3:**
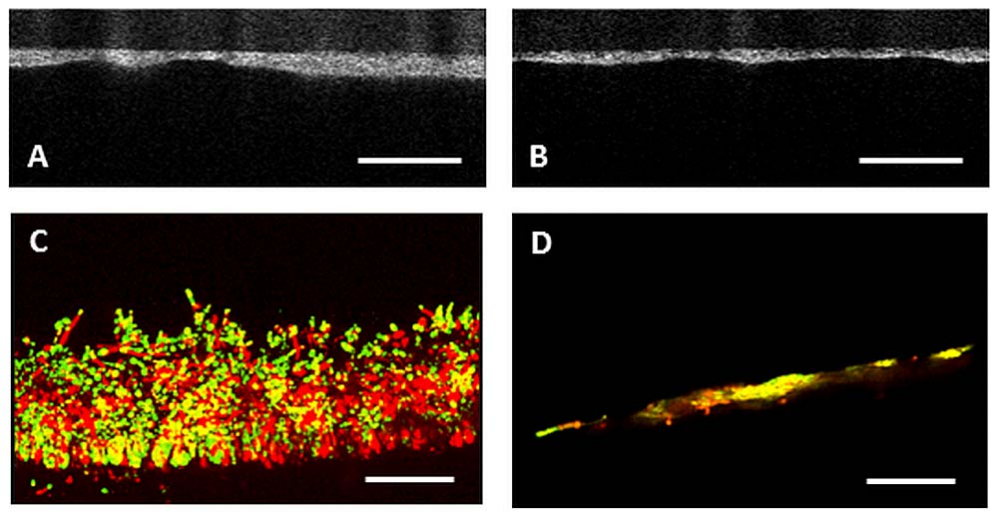
Visualization of mixed species biofilms on silicone-rubber tubes obtained using OCT and CLSM. A. In situ cross section of a mixed species biofilm of *C. tropicalis* combined with *L. crispatus* by OCT. Average biofilm thickness is 80±24 µm. In situ cross section of a mixed species biofilm of *C. albicans* combined with *R. dentocariosa* by OCT. Average biofilm thickness is 26±8 µm. B. A cross section of a mixed species biofilm of *C. tropicalis* combined with *L. crispatus* by CLSM after live/dead staining. Average biofilm thickness is 109±29 µm. C. A cross section of a mixed species biofilm of *C. albicans* combined with *R. dentocariosa* by CLSM after live/dead staining. Average biofilm thickness is 7± µm. The bars in A and C denote 500 µm, while in B and D bars represent 75 µm.

Examples of OCT and CLSM images of biofilms grown with different combinations of yeast and bacteria are shown in Figure 3. Note that biofilms consisting of a combination of *C. tropicalis* with *L. crispatus* are thicker than observed for a combination of *C. albicans* with *R. dentocariosa* regardless of the technique applied, although biofilms thicknesses obtained using OCT do not match numerically with those from CLSM. This is most likely related with the fact that tubes with biofilm were sectioned for CLSM, while OCT was done on in situ biofilms. Moreover, CLSM requires staining.

Table 1 summarizes the total numbers of CFU/cm^2^ of the mixed species biofilm on the silicone-rubber tubes. In general, biofilms consisting of combinations of *C. albicans* with a bacterial strain comprised significantly less viable organisms than *C. albicans* biofilms grown in the absence of bacteria. *C. tropicalis* on the other hand, showed a higher number of viable organisms when grown in combination with a bacterial strain, except when grown in combination with *S. salivarius*. The percentage of *Candida* present in biofilms grown in combination with *lactobacilli*, was systematically higher than when grown in combination with other commensal bacterial strains. In general, biofilms comprising *C. tropicalis* harvested higher numbers of viable organisms than when *C. albicans* was involved, in line with the biofilm thicknesses observed using OCT and CLSM (compare Figure 3 and Table 1).

In *C. albicans* biofilms however, growth in presence of *R. dentocariosa* significantly stimulated hyphal formation (see Table 2), while especially *L. casei* significantly suppressed morphogenic conversion of *C. albicans* to its hyphal form. In *C. tropicalis* biofilms, we did not see a significant stimulation by *R. dentocariosa* on hyphal formation, while *L. casei* reduced hyphal formation.

**Table 2:** Percentage hyphae occurring in *Candida* biofilms grown in the absence or presence of selected bacterial strains on silicone-rubber in an eight day time period.

*Candida* combined with	*C. albicans* (%hyphae)	*C. tropicalis* (%hyphae)
No bacteria	0.7^a^	3.0
*R. dentocariosa*	1.9±0.8^b^	2.1±1.8
*L. casei*	0.2±0.1^b^	1.6±1.3
*L. acidophilus*	0.7±0.3^a^	2.0±1.4
*L. crispatus*	0.5±0.2^a^	2.6±3.0

During the growth period, biofilms were exposed to nutritional feast and famine. All results are from triplicate experiments with separate microbial cultures and are presented ± SD. Data are normalized with respect to the average percentage of hyphae in *Candida* biofilms grown in absence of bacteria. a≠b at p<0.05.

## Discussion

The clinical life-time of silicone-rubber voice prostheses varies over extremely wide ranges within different laryngectomized patients. Sub-division of 692 failed voice prostheses into a short lifetime group (implantation-period less than 4 months) and an extended lifetime group (implantation-period over 9 months) revealed that *R. dentocariosa* and *C. albicans* and *C. tropicalis* were predominant strains in the short lifetime group. In the extended lifetime group *R. dentocariosa* was found with a fourfold lower isolation frequency and *C. albicans* was found with a twofold lower isolation frequency [3]. In a separate study, it was shown that daily consumption of a fermented milk containing *L. casei* yielded an increased prosthesis lifetime by a factor of 3.76 as compared to a historical control in the same group of patients [13].

**Figure 4:**
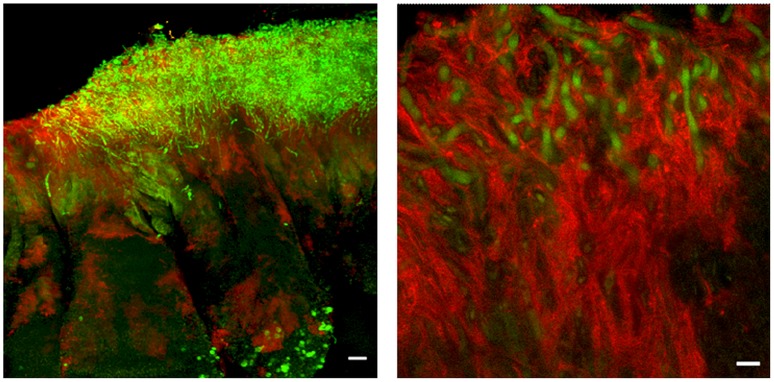
Association between yeast and lactobacilli in an in vivo formed biofilm on a voice prosthesis. Overlay-images of a biofilm from an explanted voice prosthesis (life time 318 days) hybridized with the FITC-labelled EUK516 probe indicating all yeasts (with hyphae) and Cy3-labelled Lab158 probe illustrating presence of lactobacilli. The high magnification panel on the right clearly shows the association of lactobacilli (red) and yeasts (green). Bars equal 20 µm and 5 µm for the left and right panels, respectively. Taken with permission from [14].

In the present study we show that these observations on the clinical life-time of voice prostheses cannot be explained on the basis of numbers of microorganisms found in dual-species biofilms on silicone-rubber, or for that matter: the biofilm thickness. Combinations of *lactobacillus* strains with *Candida* yield less biofilm, but with an increased prevalence of *Candida* as compared with combinations involving other bacterial strains. At the same time, we show that *L. casei*, with demonstrated favorable effects on the clinical life-time of voice prostheses [13] and adhering in full grown clinical biofilms in close association with yeast (see Figure 4), reduced the percentage hyphal formation in *Candida* biofilms as compared with *Candida* biofilms grown in absence of bacteria by a factor of three. When compared with the percentage hyphal formation in *Candida* biofilms grown in combination with *R. dentocariosa*, a bacterial strain whose presence is associated with short clinical life-times of voice prostheses [3], this factor becomes ten. Therewith we here demonstrate that in voice prosthetic biofilms, morphogenic conversions in *Candida* biofilms depend on the bacterial strain present, corresponding with effects on the clinical life-time of the prostheses.

The percentage hyphae in *Candida* biofilms grown in absence of bacteria amounts 0.7% to 3.0% (see Table 2). Other studies, in which *Candida* biofilms were grown in serum demonstrate much higher percentages of hyphae up to 100%, which is due to the hyphal transforming activity in serum associated with the signaling molecules prostaglandin and thromboxane [15]. Also the periods of nutritional feast and famine applied in the current study to mimic the conditions in the oropharynx more closely, may have a reducing effect on morphogenic conversions in *Candida*. More importantly, interactions between *Candida* and *lactobacilli* have been described to regulate *Candida* morphogenesis and therewith its virulence and invasiveness. Short chain fatty acids produced by different strains of *lactobacilli* (*L. casei*, *Lactobacillus paracasei* and *Lactobacillus rhamnosus*) inhibited hyphal formation, as well as culture supernatants of *lactobacilli* and live *lactobacilli* [15]. In the present study, we only find a significant reduction in *C. albicans* hyphal formation for *L. casei* with respect to *Candida* growth in absence of bacteria, while for *C. tropicalis* reductions observed are not statistically significant. For *C. albicans* however, the percentage hyphal formation when grown in presence of *L. casei* is tenfold lower (statistically significant at p<0.05) than when grown in presence of *R. dentocariosa*. A similar comparison exists for *C. tropicalis* but is not statistically significant.

Thus we conclude that *R. dentocariosa* stimulates morphogenic conversion in *Candida* toward a hyphal morphology, coinciding with short clinical life-times of voice prostheses from which *R. dentocariosa* was isolated [3]. In addition it is concluded that especially *L. casei* reduces hyphal formation in *Candida*, which coincides with clinical observations that the consumption of a fermented milk with *L. casei* extends the clinical life-time of voice prostheses in laryngectomized patients [13]. These conclusions are based on relatively small differences in hyphal formation. However, these differences were established over a time period of only eight days, whereas the above clinical observations were made over a period of several months to years. Likely, larger effects will develop over time. Moreover, a small number of hyphae may be sufficient to grow into the silicone-rubber and the close proximity of *Candida* and bacteria in pockets created in silicone-rubber [16] will enhance the effects of bacterial presence on morphogenic conversion of *Candida*. Since hyphal ingrowth is a major factor in the strong binding of initial *Candida* biofilms to silicone-rubber [16], [17], biofilm maturation after initial adhesion does not necessarily have to involve *Candida* in its hyphal morphology. This is in line with the high percentages of *Candida* in biofilms grown in the presence of *lactobacilli*, despite low conversions to the hyphal morphology (compare Tables 1 and 2).
